# Role of the Gut Microbiota in Glucose Metabolism During Heart Failure

**DOI:** 10.3389/fcvm.2022.903316

**Published:** 2022-07-04

**Authors:** Pei Bao, Zhiwei Zhang, Yixiu Liang, Ziqing Yu, Zilong Xiao, Yucheng Wang, Yong Yu, Wen Liu, Xueying Chen, Zhenzhen Huang, Yangang Su, Ruizhen Chen, Junbo Ge

**Affiliations:** ^1^Department of Cardiology, Shanghai Institute of Cardiovascular Diseases, Zhongshan Hospital, Fudan University, Shanghai, China; ^2^Reproductive Medical Center, Zhongshan Hospital, Fudan University, Shanghai, China; ^3^Key Laboratory of Viral Heart Diseases, Department of Cardiovascular Diseases, Ministry of Public Health, Shanghai Institute of Cardiovascular Diseases, Zhongshan Hospital, Fudan University, Shanghai, China

**Keywords:** gut microbiota, heart failure, glucose metabolic, 16S rRNA, glucagon

## Abstract

**Background:**

Blood glucose disorders are prevalent in heart failure, while the influence of the gut microbiota on this process remains unclear. Here, we used heart failure model mice and fecal microbiota transplantation (FMT) mice to evaluate the effect of the gut microbiota on the regulation of blood glucose during heart failure.

**Methods:**

Thoracic aortic constriction (TAC) surgery was performed in a heart failure model, while an antibiotic cocktail was used to eliminate the microbiota to establish a germ-free (GF) model. Blood glucose, insulin, and glucagon levels were measured, and an intraperitoneal glucose tolerance test (IPGTT) was performed. 16S rRNA sequencing and metabolomics were used to evaluate the changes in gut microbiota structure and metabolism induced by TAC. Another group of FMT mice was established to observe the effect of the gut microbiota on host metabolism.

**Results:**

After microbiota clearance, the glucagon concentration, the homeostasis model assessment for insulin resistance (HOMA-IR), and the area under the curve (AUC) of the IPGTT were decreased significantly in the TAC germ-free (TAC-GF) group in the third month as compared to the other groups. 16S rRNA sequencing indicated that TAC surgery affected the gut microbiota structure, and fecal metabolomics suggested that noradrenaline and adrenaline levels were higher in the TAC group than in the sham group. The FMT mice transplanted with the feces of the TAC (FMT-TAC) mice displayed a higher AUC of IPGTT, accompanied by a higher glucagon level, insulin level, and HOMA-IR than those of the mice in the other groups. The serum metabolomics of the FMT-TAC group showed that noradrenaline levels were significantly higher than those of the FMT-sham group.

**Conclusion:**

The gut microbiota and its metabolism were altered during heart failure, which increased blood glucose and glucagon in the host.

## Graphical Abstract

Heart failure affects microbiota structure and metabolism, and alterations in the gut microbiota play a role in regulating host blood glucose. 
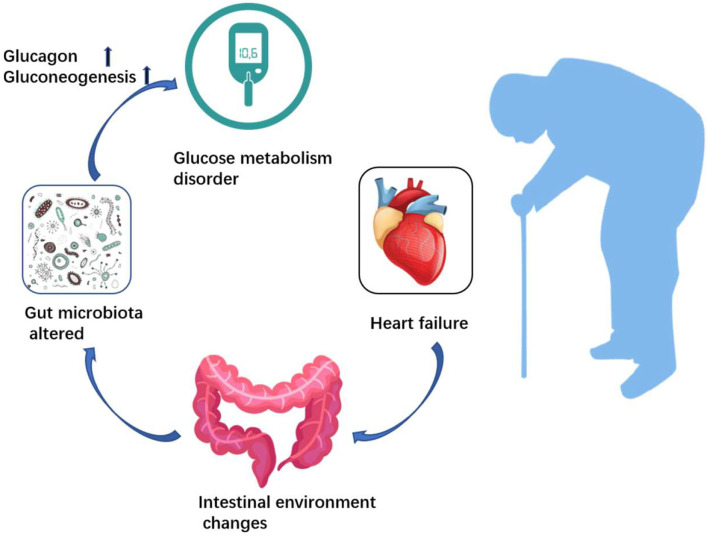

## Introduction

Epidemiological investigations indicate that patients with heart failure are more prone to develop insulin resistance and diabetes than normal individuals (1, 2). As an independent risk factor, hyperglycemia is correlated with high morbidity and hospitalization in patients with heart failure (3, 4). Therefore, there is a growing need to verify the association between heart failure and blood glucose disorder and the underlying mechanisms.

The gut microbiota is known to be closely associated with hyperglycemia and diabetes (5, 6). Many studies have shown impaired glucose tolerance in mice transplanted with feces from insulin-resistant patients, (7, 8) which indicates that the host can affect the microbiota structure; on the other hand, gut microbiota dysbiosis impairs the insulin sensitivity and glucose homeostasis of the host. Therefore, there are bidirectional interactions between the gut microbiota and host when insulin resistance occurs.

Compositional and functional alterations in the gut microbiota have been investigated in patients with heart failure. Changes in the intestinal environment induced by peripheral ischemia may be the leading cause of the imbalance in the microbiota and their metabolites (9, 10). However, whether and how the reconstitution of the gut microbiota is involved in the development of heart failure remain obscure.

To address these questions, we established a thoracic aortic constriction (TAC) mouse model and performed 16S rRNA sequencing and metabolomics analysis. Furthermore, fecal microbiota transplantation (FMT) was performed to explore the potential functions of the transferred microbiota by measuring blood glucose and metabolomics.

## Methods

### Heart Failure Mouse Model Establishment

Mice subjected to TAC surgery were used to generate a pressure overload-induced heart failure model, and the details of the surgery were similar to those in Rockman's study (11). Male C57BL/6J specific pathogen-free (SPF) mice (6–8 weeks old) weighing 20–25 g were purchased from JSJ Company (Grand Haven, MI, USA). All mice were housed in an SPF environment with a 12-h light/a12-h dark cycles and were fed sterile food (SWS9102, Xie Tong) and water *ad libitum*. Two weeks later, the animals were randomized into two groups for either TAC or sham surgery. Each month after surgery, cardiac function was assessed by a transthoracic echo-cardiographic examination and measurement of brain natriuretic peptide (BNP). The BNP level was measured with an ELISA kit (E-EL-MO2O4c, Elabscience). Cardiac fibrosis was determined by Masson's Trichrome Staining (G1340, Solarbio).

The animal experiment was approved by the Animal Ethics Committee of Shanghai Medical College, Fudan University.

### Germ-Free (GF) Mouse Establishment

**Figure 1:**
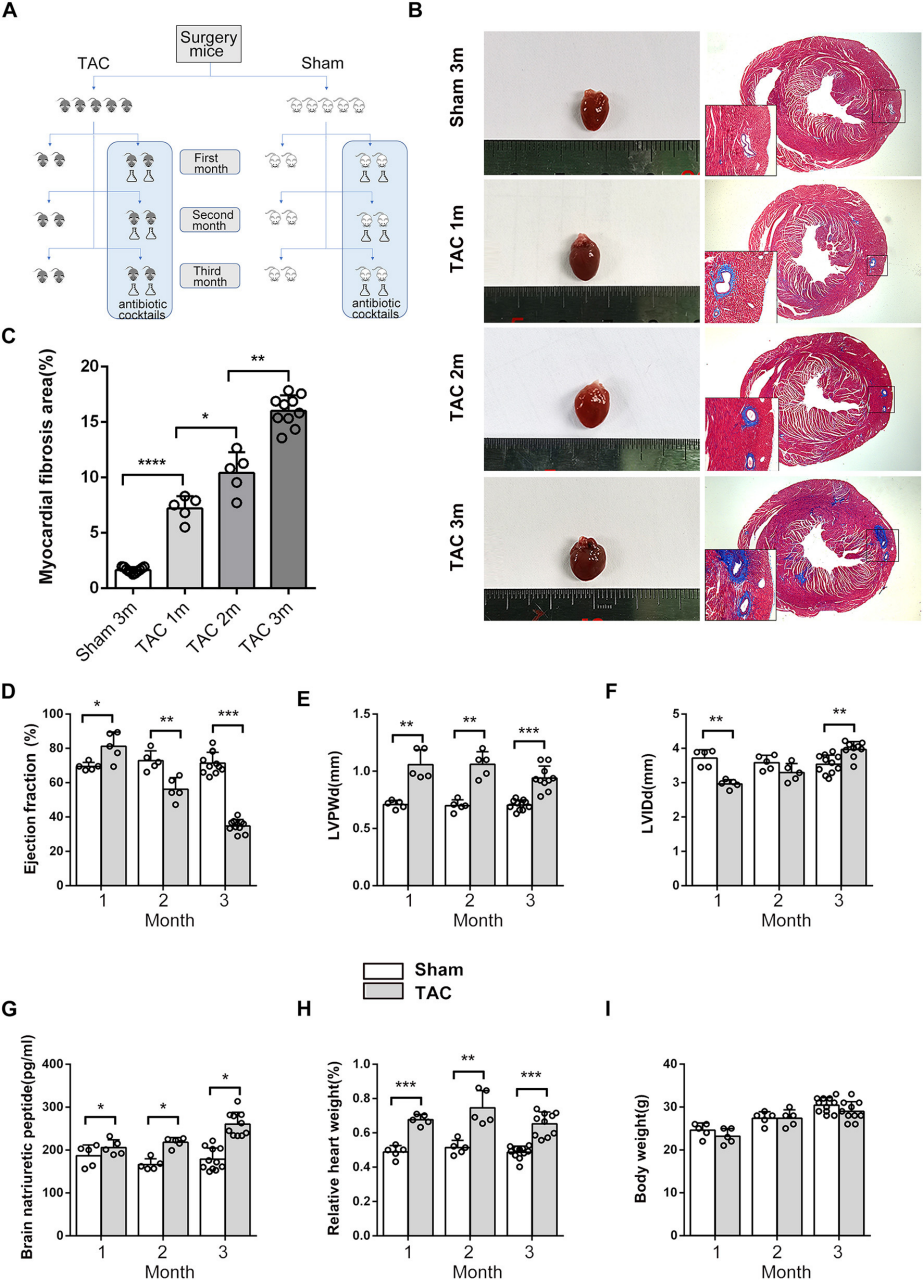
Cardiac morphometry and function during the 3 months after surgery. **(A)** Schematic diagram of the study design. **(B,C)** Cardiac size and cardiac fibrosis as shown by Masson's Trichrome Staining. **(D)** Heart function was determined based on transthoracic echocardiographic examination and ejection fraction (EF). **(E)** Left ventricular posterior wall dimensions at end-diastole (LVPWd). **(F)** Left ventricle end-diastolic dimensions. **(G)** Serum brain natriuretic peptide (BNP) levels. **(H)** Relative HW. **(I)** Body weight (BW). *n* = 5–11. ^*^p < 0.05, ^**^p < 0.01, ^***^p < 0.001.

To evaluate the role of the microbiota in blood glucose homeostasis, each month after the surgery, five mice were randomly selected from the TAC and the sham surgical groups and treated with antibiotic cocktails for 10 days (antibiotics were stopped for the last 3 days) to produce TAC germ-free (TAC-GF) mice and sham-GF mice, as shown in Figure 1A. To establish the GF mouse model, mice were given an antibiotic cocktail (MedChemExpress; containing 100 mg/ml neomycin, 50 mg/ml streptomycin, 100 mg/ml ampicillin, 50 mg/ml vancomycin, 100 mg/ml metronidazole, 1 mg/ml bacitracin, 125 mg/ml ciprofloxacin, and 100 mg/ml ceftazidime) in their drinking water.

### Measurement of Blood Glucose

Blood glucose was measured monthly using a glucose meter (Accu-Chek222, Roche) in five mice each from the TAC-GF and the sham-GF groups and the matched mice in the TAC and the sham groups. An intraperitoneal glucose tolerance test (IPGTT) was performed after 8 h of fasting. Mice were injected with 1 g/kg glucose, and blood was taken by tail blood sampling at 0, 15, 30, 60, and 120 min after glucose injection to measure the glucose levels. Fasting insulin and glucagon levels were measured by using ELISA kits (CEA448Mu, CEB266Mu, Cloud-Clone). These assays were carried out according to the manufacturer's instructions. Insulin resistance was estimated by homeostasis model assessment for insulin resistance (HOMA-IR), defined as [fasting glucose (mmol/L) × fasting insulin (mIU/L)/22.5].

### Measurement of Intestinal and Fecal Factors

The cecum and cecal contents were collected at the time of sacrifice. Intestinal mucins were detected by Alcian blue and periodic acid-Schiff (AB/PAS) staining (G1285, Solarbio). Following the manufacturer's instructions, AB staining was performed first (at pH 2.5) for 10–20 min, followed by periodic acid-Schiff staining. Fecal lactate levels were measured by a colorimetric assay kit (A019-2-2, Jiancheng). The fecal wet-to-dry ratio was defined as (wet weight–dry weight)/dry weight. Fecal noradrenaline and adrenaline levels were measured by targeted high-performance liquid chromatography mass spectrometry (HPLC-MS; AB ExionLC, AB Sciex Qtrap 6500+, AB Sciex).

### Fecal Microbiota Transplantation

Fresh fecal samples from the donor mice were suspended in phosphate-buffered saline (PBS) buffer after 10 min of vortexing (1 ml of PBS was added per 100 mg of feces) and were then centrifuged for 1 min at 500 × *g*. The supernatant was pipetted for gavage, and all procedures were performed within 4 h. The feces used for transplantation were obtained from the mice in the surgery group in the third month. Twenty-four conventional GF mice were prepared for transplantation and were randomized to receive FMT from either the TAC (FMT-TAC) or the TAC-GF (FMT-TG) group or the sham (FMT-sham) or the sham-GF (FMT-SG) group in a one-to-one correspondence (i.e., one donor to one recipient). Each GF mouse was administered 200 μl of fecal sample suspension by oral gavage two times per week for the first 2 weeks and weekly thereafter for a total of 4 weeks, and the colonization was confirmed by 16S rRNA sequencing.

### DNA Extraction and 16S rRNA Sequencing

The fecal samples of the remaining mice in the sham and the TAC groups were collected and stored at −80°C for 16S rRNA sequencing. The gut microbiota DNA concentration and integrity were determined with a NanoDrop 2000 Spectrophotometer (Thermo Fisher Scientific, Waltham, MA, USA) and agarose gel electrophoresis, respectively. PCR amplification of the V3–V4 hypervariable regions of the bacterial 16S rRNA gene was carried out in a 25-μl reaction using a universal primer pair (343F: 5′-TACGGRAGGCAGCAG-3′; 798R: 5′-AGGGTATCTAATCCT-3′). Sequencing was performed on an Illumina MiSeq (Illumina Inc., San Diego, CA, USA) with two paired-end read cycles of 300 bases each.

### Bioinformatics Analysis

The FASTQ format raw data were preprocessed using Trimmomatic software. Sequences were further denoised with QIIME software (version 1.8.0). Clean reads were subjected to primer sequence removal and clustering to generate operational taxonomic units (OTUs) using VSEARCH software with a 97% similarity cutoff. The representative read of each OTU was selected using the QIIME package. All representative reads were annotated and blasted against the Silva database version 138 (16S/18S rDNA) using the Ribosomal Database Project (RDP) classifier. Principal component analysis (PCA) and principal coordinate analysis (PCoA) were conducted by using the R language. To characterize microbial functions, we annotated all of the genes in our catalog to the Kyoto Encyclopedia of Genes and Genomes (KEGG) database.

### Untargeted Metabolomic Profiling of Fecal and Serum Samples

Untargeted metabolomics profiling of fecal and serum samples was performed. Briefly, 40 mg of each fecal sample from the surgical mice or 80 μl of each serum sample from the FMT mice was extracted with methanol before lyophilization. Then, 15 mg/ml of methoxylamine hydrochloride in pyridine was subsequently added and incubated at 37°C for 90 min. Bis (trimethylsilyl)trifluoroacetamide (BSTFA; with 1% trimethylchlorosilane) and n-hexane were added to the mixture, which was derivatized at 70°C for 60 min. The samples were placed at ambient temperature for 30 min. The derivatized samples were analyzed on an Agilent 7890B Gas Chromatography System coupled to an Agilent 5977AMSD System (Agilent Technologies Inc, CA, USA).

### Metabolomic Data Analysis

The metabolomics data were converted by Abf Converter and analyzed by MS-DIAL. Metabolite characterization was based on the LUG database (Untargeted database from Lumingbio). In each sample, all peak signal intensities were segmented and normalized according to the internal standards with relative standard deviations (SDs) >0.3 after screening to obtain the data matrix. The matrix was imported into R to carry out a partial least-squares-discriminant analysis (PLS-DA) to distinguish the metabolites that differed between groups. To prevent overfitting, 7-fold cross-validation and 200 response permutation testing (RPT) were used to evaluate the quality of the model. A two-tailed Student's *t*-test was further used to verify whether the differences in metabolites between groups were significant. Metabolites with variable importance in the projection (VIP) values >1.0 and *p*-values <0.05 were selected as differential metabolites. Allocation of the metabolites within designated pathways was supported by KEGG.

### Statistical Analysis

Statistical analysis was performed using SPSS (version 25) software. Continuous variables are presented as the mean ± standard error of the mean (SEM). Two-group comparisons were performed using Student's non-paired *t-*test or the Wilcoxon (Mann-Whitney U) test. Variables with more than two groups were analyzed by a one-way ANOVA. The statistics regarding the microbiome subsection analyses are described in the gut microbiota profiling section of “Methods.” Correlations were calculated with the Spearman rank correlation coefficient. A level of *p* < 0.05 was considered statistically significant.

## Results

### Gut Microbiota Effects on Blood Glucose

**Figure 2:**
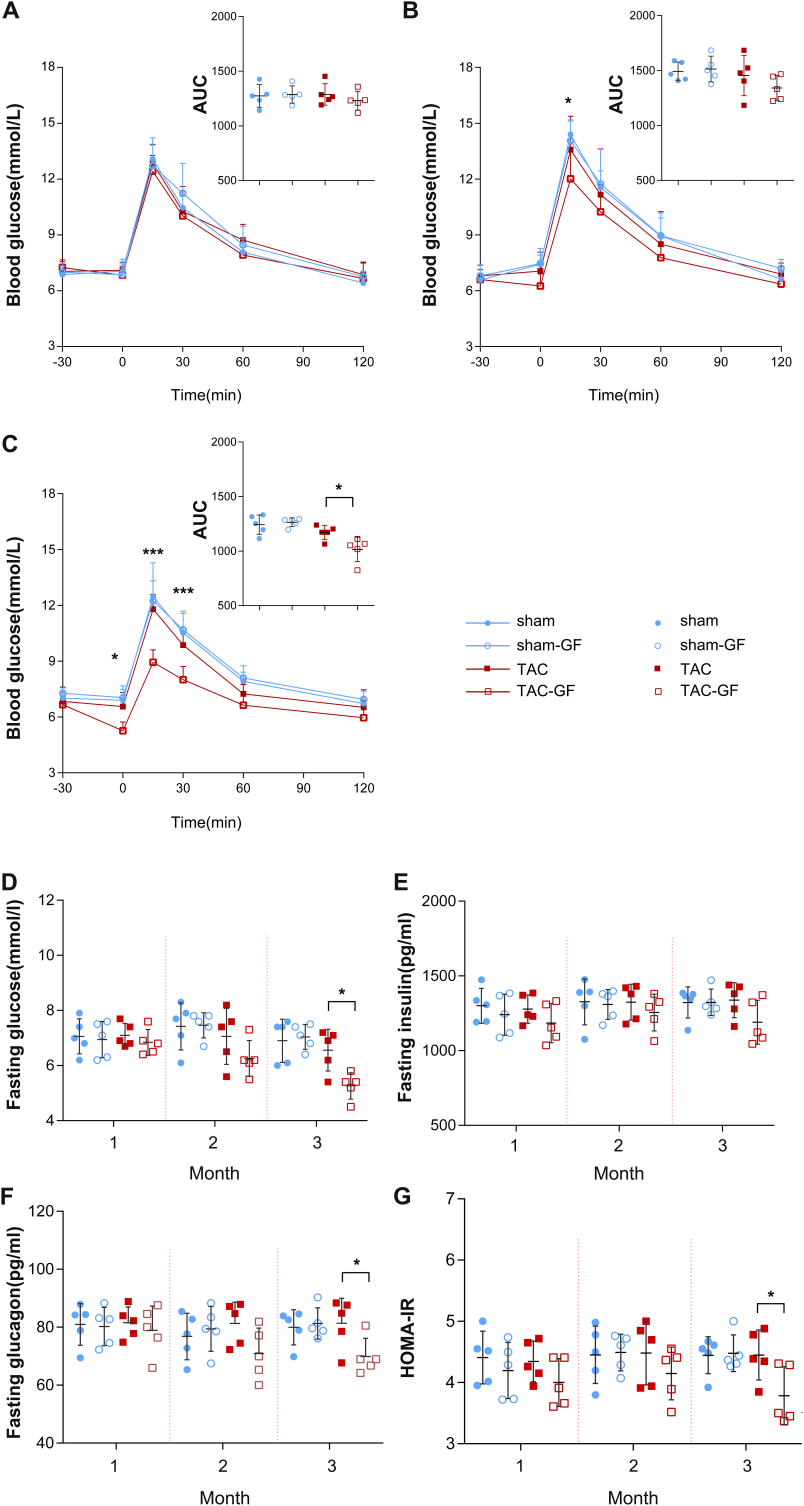
The gut microbiota influence glucose tolerance after thoracic aortic constriction (TAC) surgery. **(A–C)** Intraperitoneal glucose tolerance test (IPGTT) performance in the TAC, TAC germ-free (TAC-GF), sham, and sham-GF groups each month after surgery. **(D)** Fasting glucose levels. **(E)** Fasting insulin levels. **(F)** Fasting glucagon levels. **(G)** Homeostasis model assessment for insulin resistance (HOMA-IR) value. *n* = 5. ^*^p < 0.05.

During the 3 months after surgery, the heart function of the TAC group decreased gradually, as shown by transthoracic echocardiography and BNP levels. At the end of the third month, all of the TAC group mice had an ejection fraction (EF) <50% and significantly increased perivascular fibrosis, left ventricular posterior wall dimensions at end-diastole (LVPWd), left ventricular internal diameter diastole (LVIDd), BNP, and heart weight (HW) (Figures 1B–H); however, the body weight (BW) of the mice was not altered significantly (Figure 1I), and antibiotics did not affect these parameters (Supplementary Figure 1). Fasting and random blood glucose, insulin, glucagon, and IPGTT levels did not show any differences between these groups in the first 2 months (Figures 2A,B), whether the microbiota was cleared or not, but in the third month after surgery, the IPGTTs of the TAC-GF mice revealed decreased glucose levels at the 15-, 30-, and 60-min time points, which did not occur in the other groups (Figure 2C). Significant decreases in the area under the curve (AUC), insulin levels, glucagon levels, and HOMA-IR were also observed in the TAC-GF group. Antibiotics did not affect blood glucose, insulin, or glucagon levels in the other groups (Figures 2D–G).

### Intestinal and Fecal Alterations

**Figure 3:**
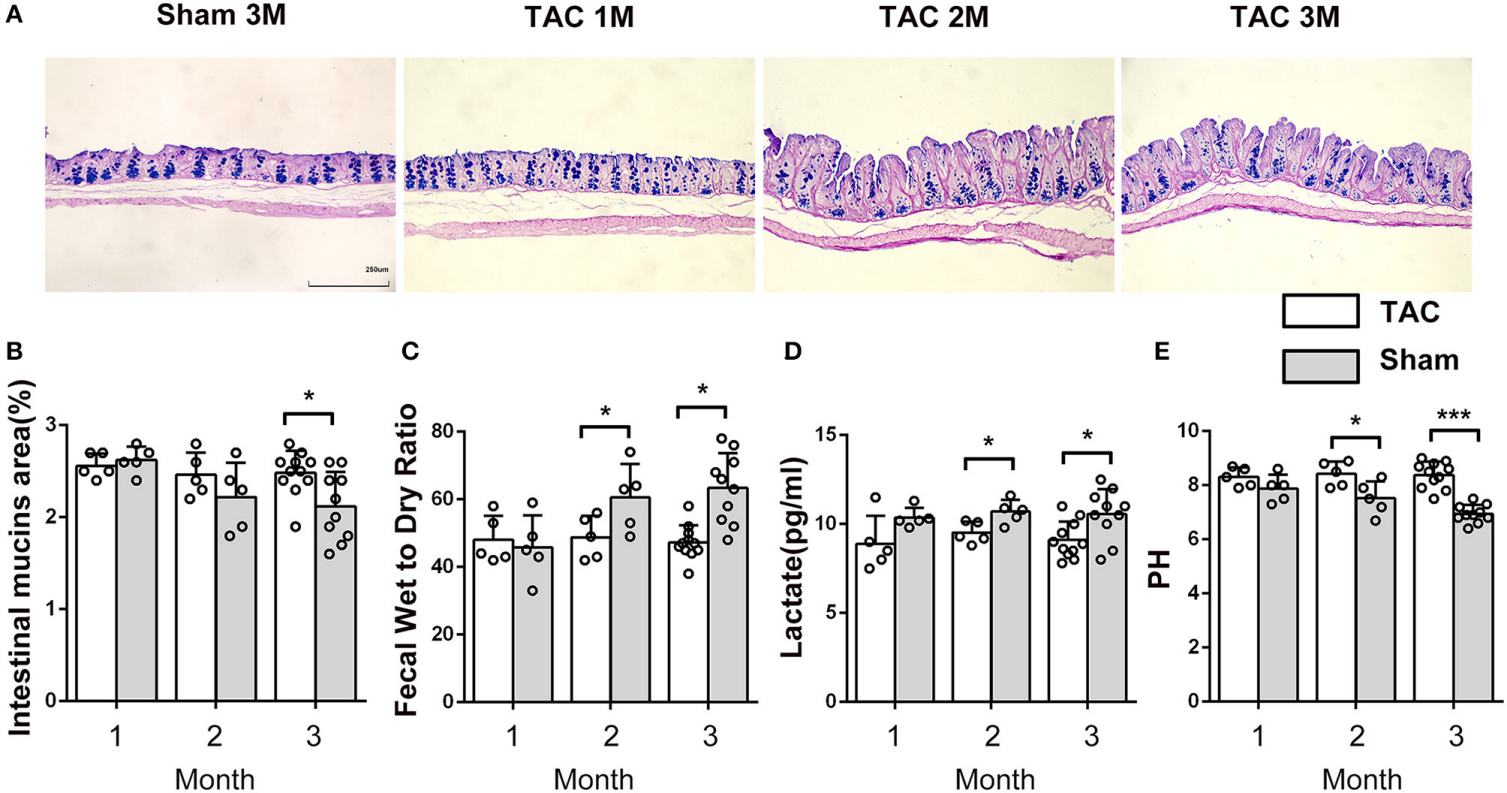
The properties of the intestine and the feces changed after thoracic aortic constriction (TAC) surgery. **(A)** Cecum Alcian blue and periodic acid-Schiff (AB-PAS) staining. **(B)** Acid mucin relative area of the TAC and sham groups. **(C)** Fecal water content in the two groups after drying at 60°C for 12 h. **(D)** Lactate level in feces. **(E)** pH of feces (100-mg fecal sample in 500 μl of saline solution). *n* = 5–11. *p < 0.05, ***p < 0.001.

Thoracic aortic constriction surgery changed intestinal and fecal functions. A decrease in intestinal acidic mucin levels was observed in the TAC group (Figures 3A,B). The feces of the TAC group had higher water and lactate contents and a lower pH value (Figures 3C–E). Antibiotics did not influence the intestinal acidic mucin levels, fecal water content, or pH value; however, they decreased the lactate levels in the feces in both the TAC-GF and the sham-GF groups (Supplementary Figure 2).

### Changes in the Gut Microbiota Structure During Heart Failure

**Figure 4:**
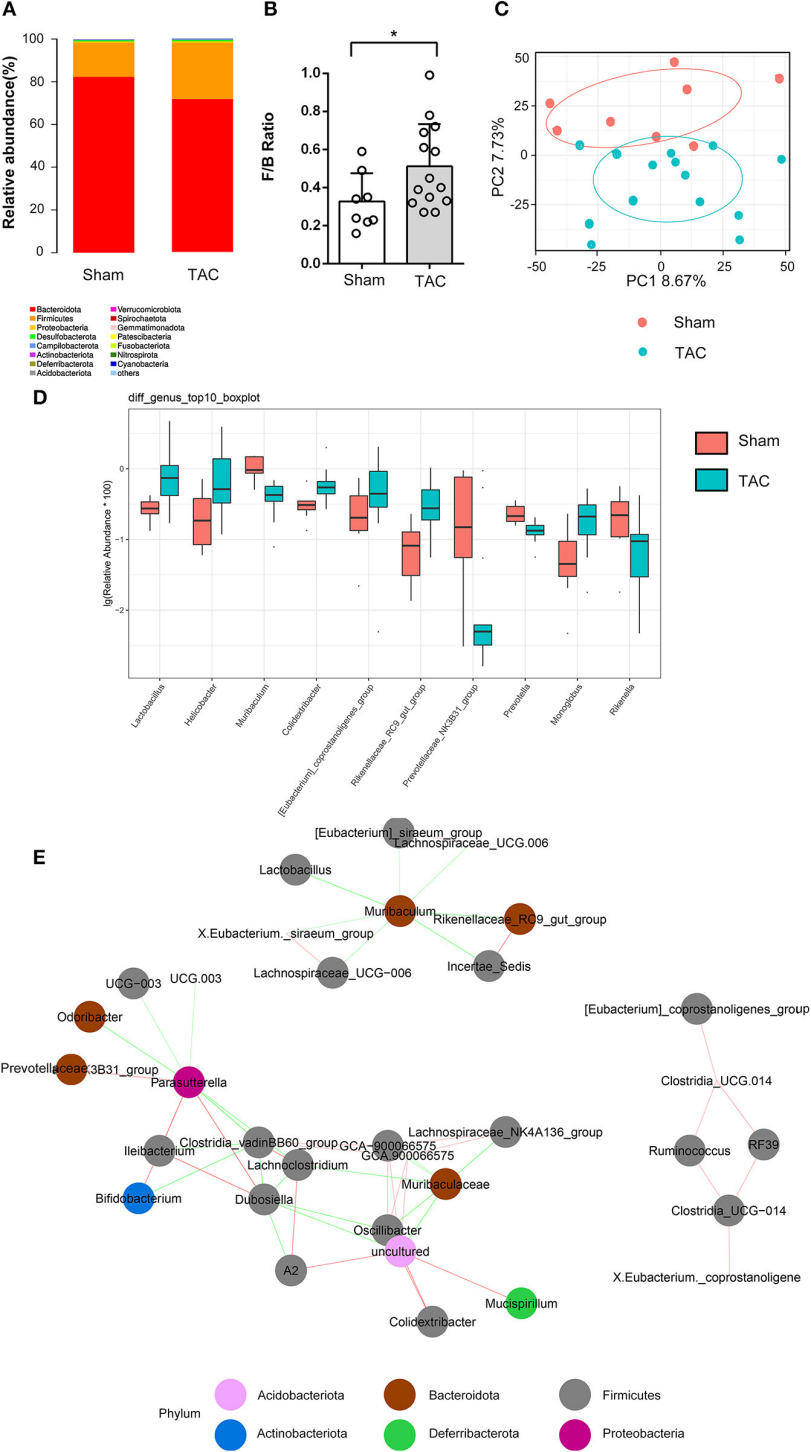
Thoracic aortic constriction (TAC) surgery altered the gut microbiota, as measured by 16S rRNA sequencing. **(A)** Microbiota constituents are reflected by the relative abundances of microbiota. **(B)** F/B ratio of the TAC and sham groups at the phylum level. **(C)** Principal component analysis (PCA) of β diversity analysis based on Bray-Curtis distances. **(D)** The top 10 significantly differentially abundant genera in the two groups. **(E)** The coabundance relationships between the differential microbiota. The color of the connecting line indicates positive and negative correlation, where red indicates a positive correlation and green indicates a negative correlation. The shade of the color represents the strength of the correlation. Circles with different colors represent different taxonomies at the phylum level.

**Figure 5:**
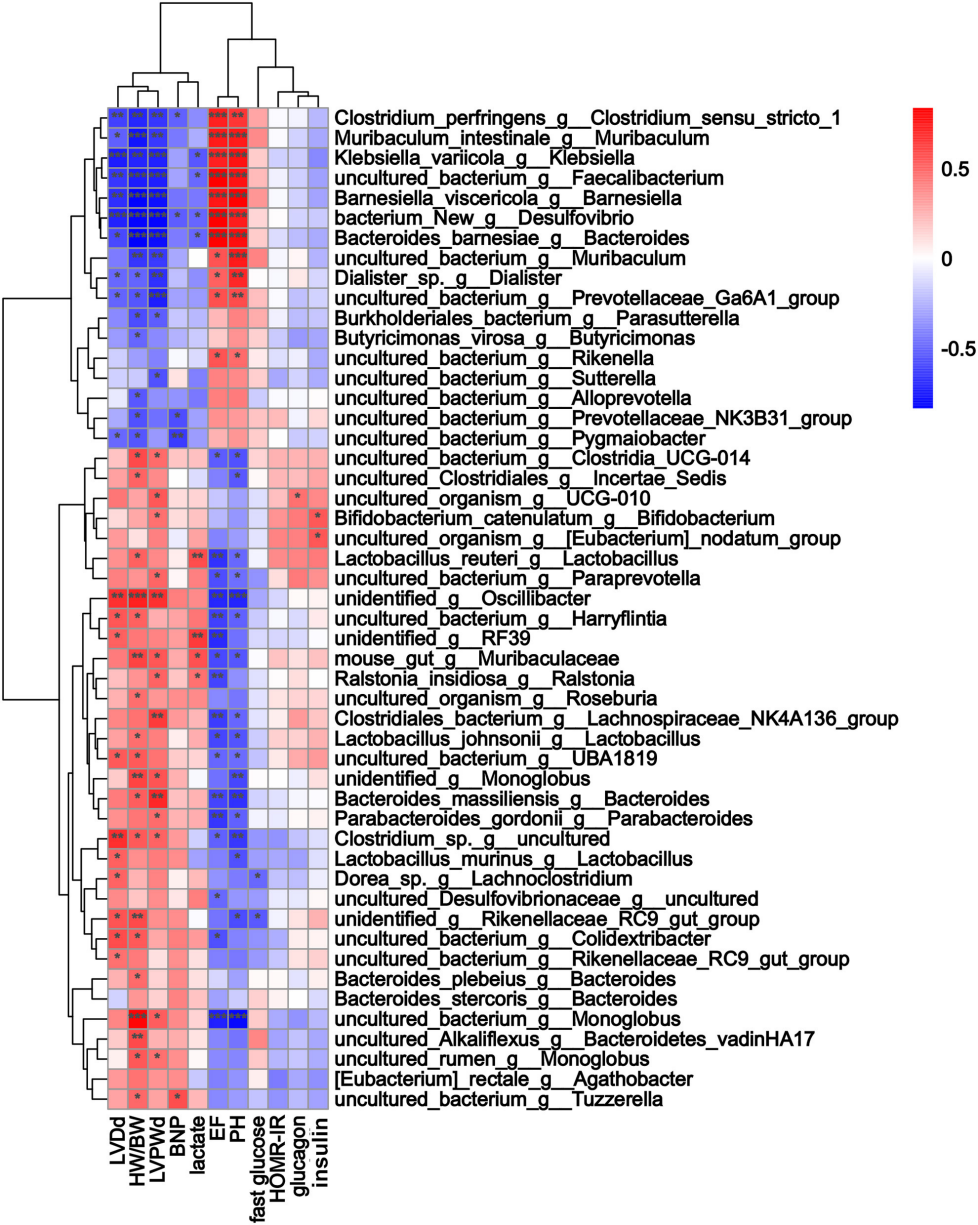
The alterations in microbial species were associated with heart function parameters. Heatmap of Spearman's correlations between changes in cardiac function, glucose levels, fecal indices, and microbiota alterations caused by thoracic aortic constriction (TAC). Red indicates a positive correlation, and blue indicates a negative correlation. *p < 0.05, **p < 0.01, and ***p < 0.001.

Three months after surgery, fecal samples from 14 TAC and 8 sham group mice were successfully collected, and 16S rRNA sequencing showed that the gut microbiota was changed after TAC surgery. The numbers of OTUs were distributed between 2,193 and 2,774 in the 22 samples. Firmicutes and Bacteroidetes were the most abundant phyla in both groups, but a higher Firmicutes to Bacteroidetes ratio (F/B) was observed in the TAC group than in the sham group (Figures 4A,B). PCA revealed that the microbiota of the two groups was completely separated from each other (Figure 4C). Among the 43 most abundant differential bacteria at the genus level, the abundances of 22 were increased in the TAC group that included Helicobacter, Rikenellaceae, Colidextribacter, and Lactobacillus, while those of Prevotellaceae, Muribaculum, and Rikenella were decreased (Figure 4D). Coabundance analysis showed that Muribaculum was negatively correlated with Lactobacillus, Lachnospiraceae, Rikenellaceae, and Eubacterium, while Parasutterella was positively correlated with Bifidobacterium and Prevotellaceae (Figure 4E). The heart function data, such as HW/BW, LVPWd, LVIDd, and BNP levels, were positively correlated with Lactobacillus, Oscillibacter, Rikenellaceae, Lachnospiraceae, and Tuzzerella. The pH value was negatively correlated with Lactobacillus and Lachnospiraceae (Figure 5).

### Microbiota Metabolism Alteration During Heart Failure

**Figure 6:**
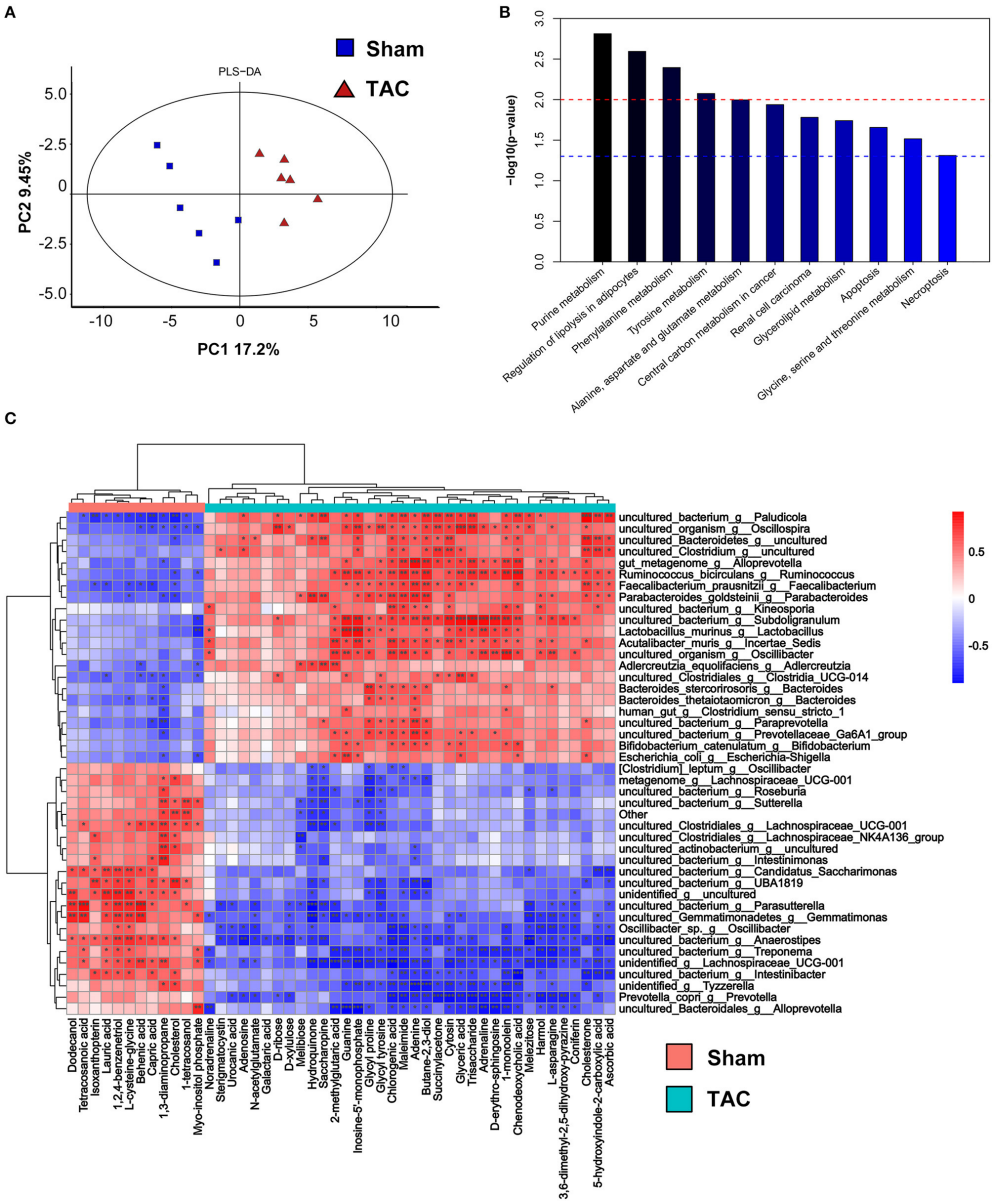
Fecal metabolomics of the surgically treated mice. **(A)** Partial least-squares-discriminant analysis (PLS-DA) of the metabolites. **(B)** The differential metabolite functions are predicted by the Kyoto Encyclopedia of Genes and Genomes (KEGG) pathway enrichment analysis. **(C)** Heatmap of Spearman's correlations between changes in metabolites and microbiota in the fecal samples. Red indicates a positive correlation, and blue indicates a negative correlation. *p < 0.05, **p < 0.01, and ***p < 0.001.

Partial least-squares-discriminant analysis indicated that the metabolites of the gut microbiota were significantly different between the TAC and the sham groups (Figure 6A). KEGG pathway enrichment showed that the metabolic alterations were enhanced in the regulation of lipolysis in adipocytes, the cyclic adenosine 3,5-monophosphate (cAMP) signaling pathway, and purine metabolism (Figure 6B). A total of 49 differential metabolites were identified. Noradrenaline and adrenaline were upregulated in the TAC group. Correlation analysis showed that noradrenaline and adrenaline were positively related to Lactobacillus and Oscillibacter (Figure 6C).

### The Gut Microbiota Affects Host Blood Glucose

**Figure 7:**
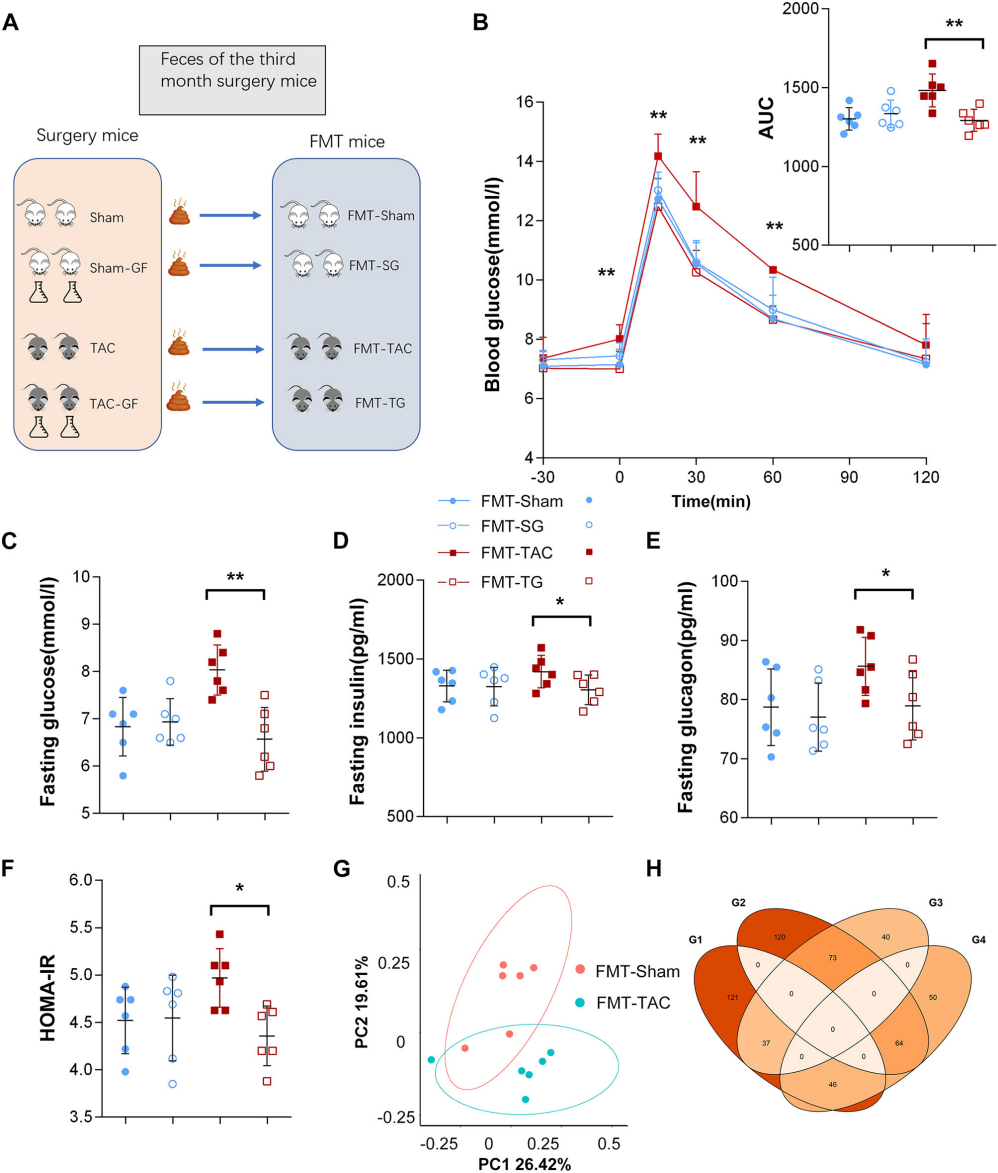
Blood glucose was influenced by fecal microbiota transplantation (FMT). **(A)** Schematic diagram of the FMT design. **(B)** Intraperitoneal glucose tolerance test (IPGTT) in the FMT from the TAC (FMT-TAC), FMT from the TG (FMT-TG), FMT-sham, and FMT-SG groups. **(C)** Fasting glucose levels. **(D)** Fasting insulin levels. **(E)** Fasting glucagon levels. **(F)** Homeostasis model assessment for insulin resistance (HOMA-IR) values. **(G)** Principal coordinate analysis (PCoA) of β diversity analysis based on Bray-Curtis distances. **(H)** Donor mice and recipient mice share the same upregulation and downregulation of microbiota. G1–G4 represent donor upregulation, donor downregulation, recipient upregulation, and recipient downregulation, respectively. *p < 0.05, **p < 0.01.

Since we found positive results in the third month after surgery and in the GF mice, we collected feces from all four groups of mice for fecal transplantation (Figure 7A). After transplantation of the TAC mouse fecal samples, the IPGTT-AUC, insulin level, glucagon level, and HOMA-IR of the FMT-TAC mice were all higher than those of the FMT-sham group mice, while the FMT mice treated with TAC-GF mouse feces (FMT-TG) and sham-GF mouse feces (FMT-SG) were not different from each other (Figures 7B–F). 16S rRNA sequencing suggested that there was a clear separation between the FMT-TAC group and the FMT-sham group, and the microbiota was similar between the donor and recipient mice (Figures 7G,H). FMT did not change the function of the heart (Supplementary Figure 3).

### The Gut Microbiota Influences Host Metabolism

**Figure 8:**
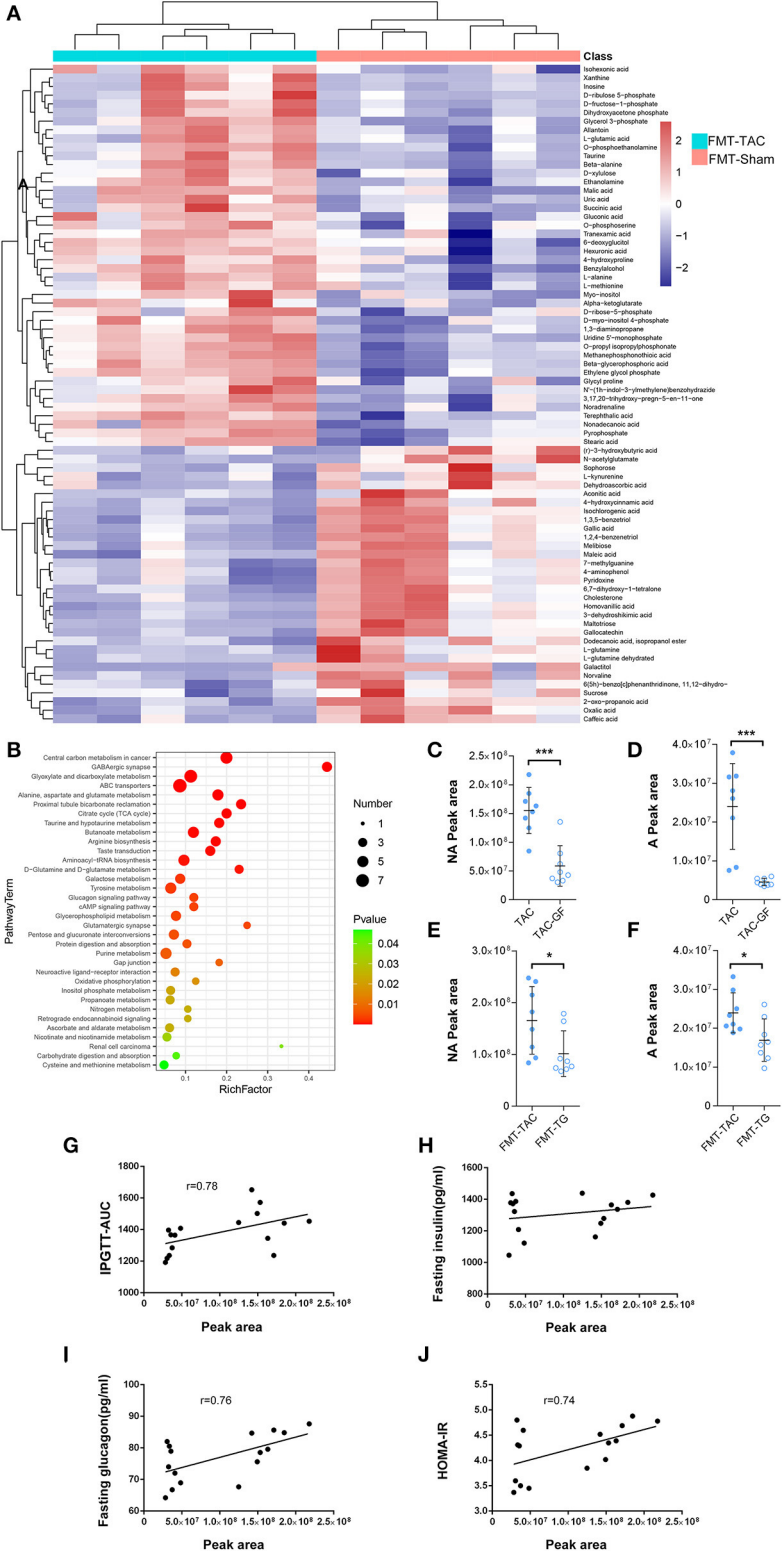
Serum metabolism was altered by fecal microbiota transplantation (FMT). **(A)** Heatmap of serum metabolism. Red indicates upregulation, and blue indicates downregulation. **(B)** The Kyoto Encyclopedia of Genes and Genomes (KEGG) pathway enrichment analysis. The color from green to red indicates a sequential decrease in the p value; the larger the dot is, the greater the number of metabolites enriched in that pathway. **(C,D)** Noradrenaline (NA) and adrenaline **(A)** levels in the fecal samples of thoracic aortic constriction (TAC) mice with or without antibiotic cocktail treatment. **(E,F)** NA and A levels in the fecal samples of FMT mice transplanted with TAC or TAC germ-free (TAC-GF) mouse samples. **(G–J)** Correlation analysis between fecal noradrenaline levels and the intraperitoneal glucose tolerance test-area under the curve (IPGTT-AUC), fasting insulin levels, fasting glucagon levels, and homeostasis model assessment for insulin resistance (HOMA-IR). *P < 0.05, ***P < 0.001.

The serum metabolism of the FMT mice showed that there were 47 metabolites enriched in the FMT-TAC group, many of which were involved in gluconeogenesis, such as beta-alanine, succinic acid, alpha-ketoglutarate, malic acid, and dihydroxyacetone phosphate (Figure 8A). The KEGG pathway enrichment analysis showed that the glucagon pathway, citrate cycle, and tyrosine metabolism were significantly enriched (Figure 8B). When comparing the serum and fecal metabolites, we found that 46 products were upregulated between fecal and serum samples, such as noradrenaline, L-glutamic acid, and serotonin. The KEGG pathway analysis indicated that these products in serum were positively correlated with the cAMP signaling pathway, pentose and glucuronate interconversions, and purine metabolism (Supplementary Figure 4).

Noradrenaline and adrenaline are normal hormones whose levels are increased during heart failure, and they may penetrate through the intestine into the feces (12). To determine the origin of noradrenaline and adrenaline, we measured noradrenaline and adrenaline in the fecal samples of TAC and TAC-GF mice. The results showed that noradrenaline and adrenaline decreased significantly in the TAC-GF mouse fecal samples, and the same result was shown in FMT-TG mice (Figures 8C–F). The correlation analysis showed that the noradrenaline level was positively correlated with the host IPGTT-AUC, glucagon level, and HOMA-IR (Figures 8G–J).

## Discussion

In this study, by employing a heart failure mouse model, we found that impaired heart function resulted in the reconstitution of the gut microbiota structure. By clearing the gut microbiota and performing FMT, we found that the microbiota plays an important role in glucagon secretion and blood glucose level increment. TAC and FMT-TAC significantly altered fecal and serum metabolites, respectively. Noradrenaline derived from the gut microbiota was correlated with glucagon and IGTT-AUC in heart failure.

Patients with chronic heart failure frequently develop blood glucose dysregulation (5, 13). Clinical studies have suggested that hyperglycemia is detrimental to heart failure (14, 15). In our study, fasting blood glucose and glucagon were decreased by eliminating gut microbiota, suggesting the hyperglycemic role of the gut microbiota, which was also validated by FMT mice. This finding suggests that the gut microbiota may be one of the factors that contribute to increased blood sugar and insulin resistance in patients with long-term chronic heart failure.

Impaired cardiac function greatly affects the gut microenvironment, (16, 17) which may contribute to altered gut microbiota structure. PCO_2_ has been reported to increase when the gastrointestinal blood supply is deficient, leading to anaerobic metabolism in the gut (18, 19). Consistent with previous studies, our results indicated that the changes in the intestinal environment with low pH and more moisture in the feces of the TAC group promoted acid stress-tolerant bacteria, such as Lactobacillus and Bifidobacterium proliferation (20, 21).

Quorum sensing may be another reason for altered gut microbiota structure during heart failure. Quorum sensing is a form of bacterial chemical communication that allows bacterial groups to synchronously change behavior in response to changes in population density and the species composition of the vicinal community (22). The coabundance analysis partially reflects the quorum sensing mechanism among the gut microbiota. The analysis showed that species that do not share similar taxa may be altered when adapting to TAC intervention, as evidenced by increased network density centralizing around the significantly altered species, such as Muribaculum, Rikenellaceae, Lactobacillus, and Oscillibacter. This finding implies that changes in the gut microbiota may not be a single microbe but a comprehensive result of changes in the structure of microbiota populations.

In recent years, several studies have demonstrated that gut dysbiosis is closely related to heart failure (23). Lactobacillus and Bifidobacterium were found to be upregulated in heart failure, (24–26) and these microbes were positively correlated with heart failure-related markers in our study. In addition, these microbes are also upregulated in diabetes, (27) suggesting that they may play a role in both heart failure and diabetes. However, our results are not identical to the gut microbiota profiles of patients with diabetes, (28, 29) suggesting that the main microbiota and mechanisms affecting blood glucose vary by disease model.

By serum metabolomics analysis, we found a marked increase in noradrenaline, serotonin, and gluconeogenic raw materials in the FMT-TAC group. Norepinephrine, which is associated with Lactobacillus and Oscillibacter, not only increases blood glucose by stimulating glucagon secretion (30) but also acts directly on the liver to stimulate gluconeogenesis (31). Increased serotonin in the peripheral circulation has also been shown to stimulate blood glucose increment and glucagon secretion (32, 33). The increase in gluconeogenic raw materials may partly reflect the enhanced glucagon pathway (34). These findings may suggest that the mechanism of blood glucose changes induced by the gut microbiota in heart failure may be the result of a variety of metabolites related to glucagon increment.

Finally, to determine the fecal metabolites responsible for the regulation of blood glucose, we analyzed the potential metabolites from gut microbes. We found that when compared with their control groups, the levels of noradrenaline in the TAC-GF group and FMT-TG group were significantly reduced with antibiotic treatment, which suggested that noradrenaline, as a regulator of blood glucose, was produced, at least partially, by gut microbiota (12, 35).

The gut microbiota has been massively reported to play a positive feedback role in many diseases, (36–38) but its contribution to comorbidities or side effects has not been intensively investigated, especially in heart failure. Our data showed that the clearance of the gut microbiota in TAC mice lowered glucagon and blood glucose, while the transfer of these microbiomes promoted hyperglycemia in healthy individuals. This investigation provides a starting view of the pathophysiological function of the gut microbiota in cardiovascular disease.

## Limitations

The metabolites that influence blood glucose are very large, and we focused on small molecules related to the gut microbiota that may exclude macromolecular substances, such as LPS, which we will discuss in future studies. As we have previously found that the microbiota structure and its metabolism displayed dynamic changes, we can infer that if we lengthened the observation period, more results would be found.

## Data Availability Statement

The datasets presented in this study can be found in online repositories. The names of the repository/repositories and accession number(s) can be found in the article/Supplementary Material. Reference for the 16S rDNA data have been deposited in the NCBI under accession code PRJNA851167 and metabolomics data have been deposited in the Metabolights under accession code MTBLS5145.

## Author Contributions

PB initiated, designed, performed the research, and wrote the manuscript. ZZ and YL designed and performed experiments. ZY, ZX, YW, YY, and WL performed experiments. XC and ZH performed date analysis. YS and RC initiated, designed, and supervised the research. JG supervised the research. All authors revised and edited the manuscript and figures. All authors contributed to the article and approved the submitted version.

## Funding

This work was funded by National Natural Science Foundation of China (82170386).

## Conflict of Interest

The authors declare that the research was conducted in the absence of any commercial or financial relationships that could be construed as a potential conflict of interest.

## Publisher's Note

All claims expressed in this article are solely those of the authors and do not necessarily represent those of their affiliated organizations, or those of the publisher, the editors and the reviewers. Any product that may be evaluated in this article, or claim that may be made by its manufacturer, is not guaranteed or endorsed by the publisher.

## Supplementary Material

Supplementary Figure 1 Cardiac morphometry and function during the 3 months after antibiotic cocktail treatment. **(A,B)** Cardiac size and cardiac fibrosis. **(C)** Ejection fraction (EF). **(D)** Left ventricular posterior wall dimensions at end-diastole (LVPWd). **(E)** Left ventricular internal diameter diastole (LVIDd). **(F)** Serum brain natriuretic peptide (BNP) levels. **(G)** Relative heart weight. **(H)** BW. *n* = 5. ^*^*p* < 0.05, ^**^*p* < 0.01, ^***^*p* < 0.001.

Supplementary Figure 2 The properties of the intestine and feces changed after surgery and GF treatment. **(A)** Cecum Alcian blue and periodic acid-Schiff (AB-PAS) staining. **(B)** Acid mucin relative area of the thoracic aortic constriction (TAC) and sham groups. **(C)** Fecal water content in the two groups. **(D)** Lactate level in feces. **(E)** pH of feces. *n* = 5. ^*^*p* < 0.05, ^**^*p* < 0.01.

Supplementary Figure 3 Fecal microbiota transplantation (FMT) did not change the morphometry and function of the cardiac tissue. **(A,B)** Cardiac size and cardiac fibrosis. **(C)** Ejection fraction (EF). **(D)** Left ventricular posterior wall dimensions at end-diastole (LVPWd). **(E)** Left ventricular internal diameter diastole (LVIDd). **(F)** Serum brain natriuretic peptide (BNP) levels. **(G)** Relative heart weight. **(H)** BW. *n* = 6.

Supplementary Figure 4 The predicted functions of the shared upregulated metabolism in fecal and serum samples. **(A)**. Shared upregulated metabolism in fecal and serum samples. **(B)** L-Glutamic acid enrichment pathway. **(C)** Noradrenaline. **(D)** Serotonin. Left, fecal; right, serum.
