# Supplementary figures and images for: Role of the Gut Microbiota in Glucose Metabolism During Heart Failure
Source: Front Cardiovasc Med. 2022 Jul 4;9:903316. doi: 10.3389/fcvm.2022.903316 (PMC9289393; doi:10.3389/fcvm.2022.903316)

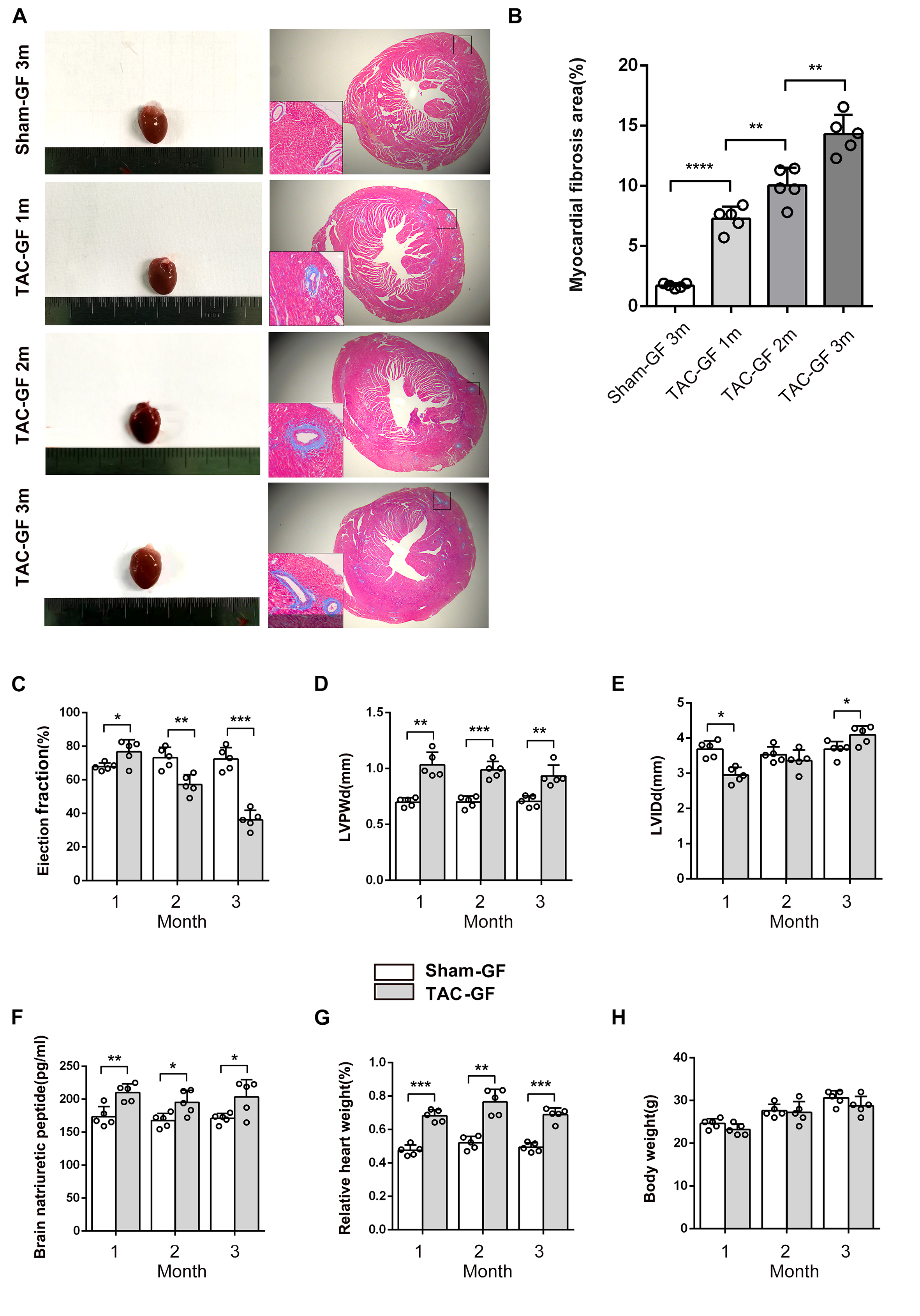

Supplement: Supplementary Figure 1 — Cardiac morphometry and function during the 3 months after antibiotic cocktail treatment. (A,B) Cardiac size and cardiac fibrosis. (C) Ejection fraction (EF). (D) Left ventricular posterior wall dimensions at end-diastole (LVPWd). (E) Left ventricular internal diameter diastole (LVIDd). (F) Serum brain natriuretic peptide (BNP) levels. (G) Relative heart weight. (H) BW. n = 5. *p < 0.05, **p < 0.01, ***p < 0.001. [file Image_1.TIF]

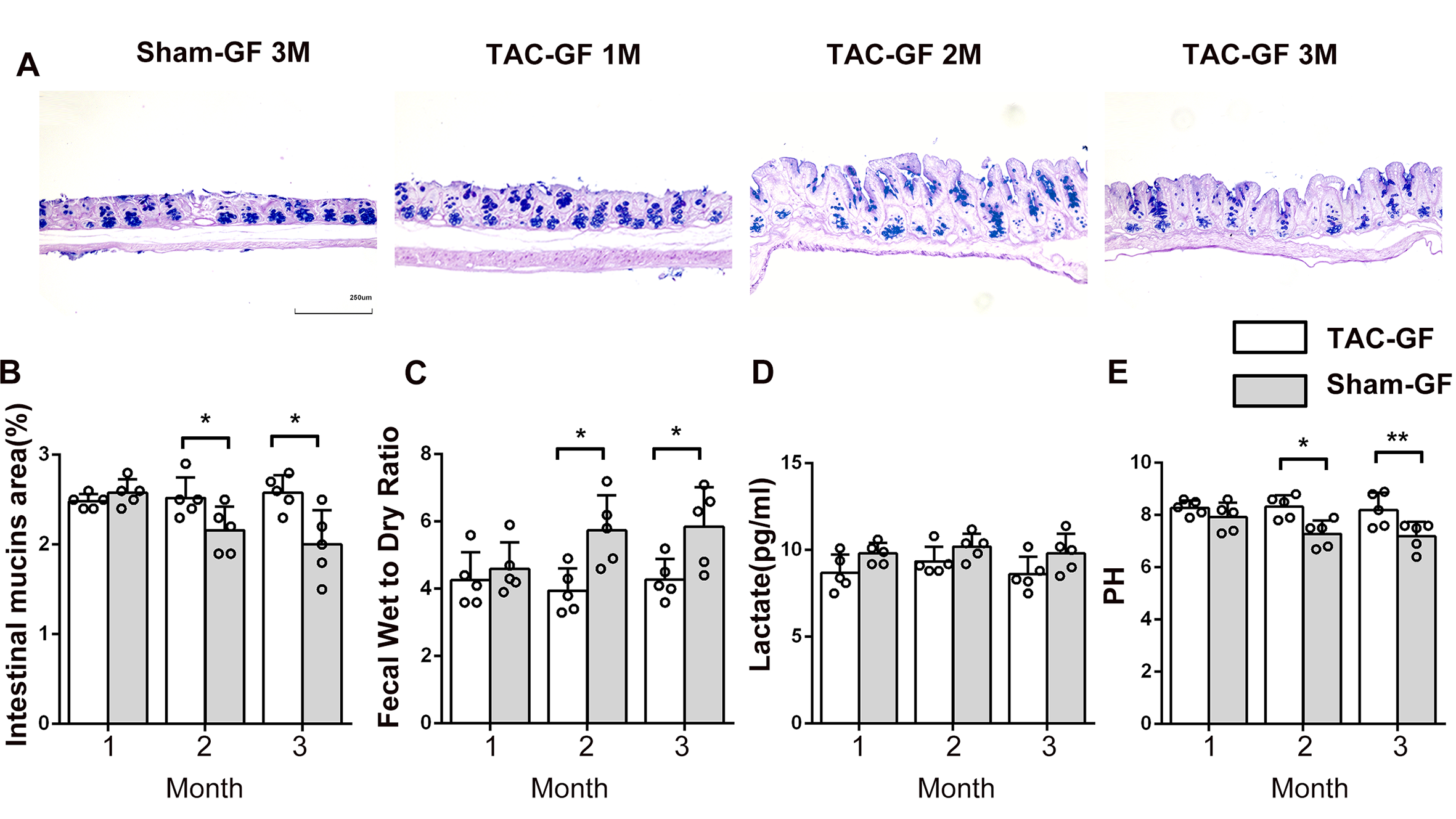

Supplement: Supplementary Figure 2 — The properties of the intestine and feces changed after surgery and GF treatment. (A) Cecum Alcian blue and periodic acid-Schiff (AB-PAS) staining. (B) Acid mucin relative area of the thoracic aortic constriction (TAC) and sham groups. (C) Fecal water content in the two groups. (D) Lactate level in feces. (E) pH of feces. n = 5. *p < 0.05, **p < 0.01. [file Image_2.TIF]

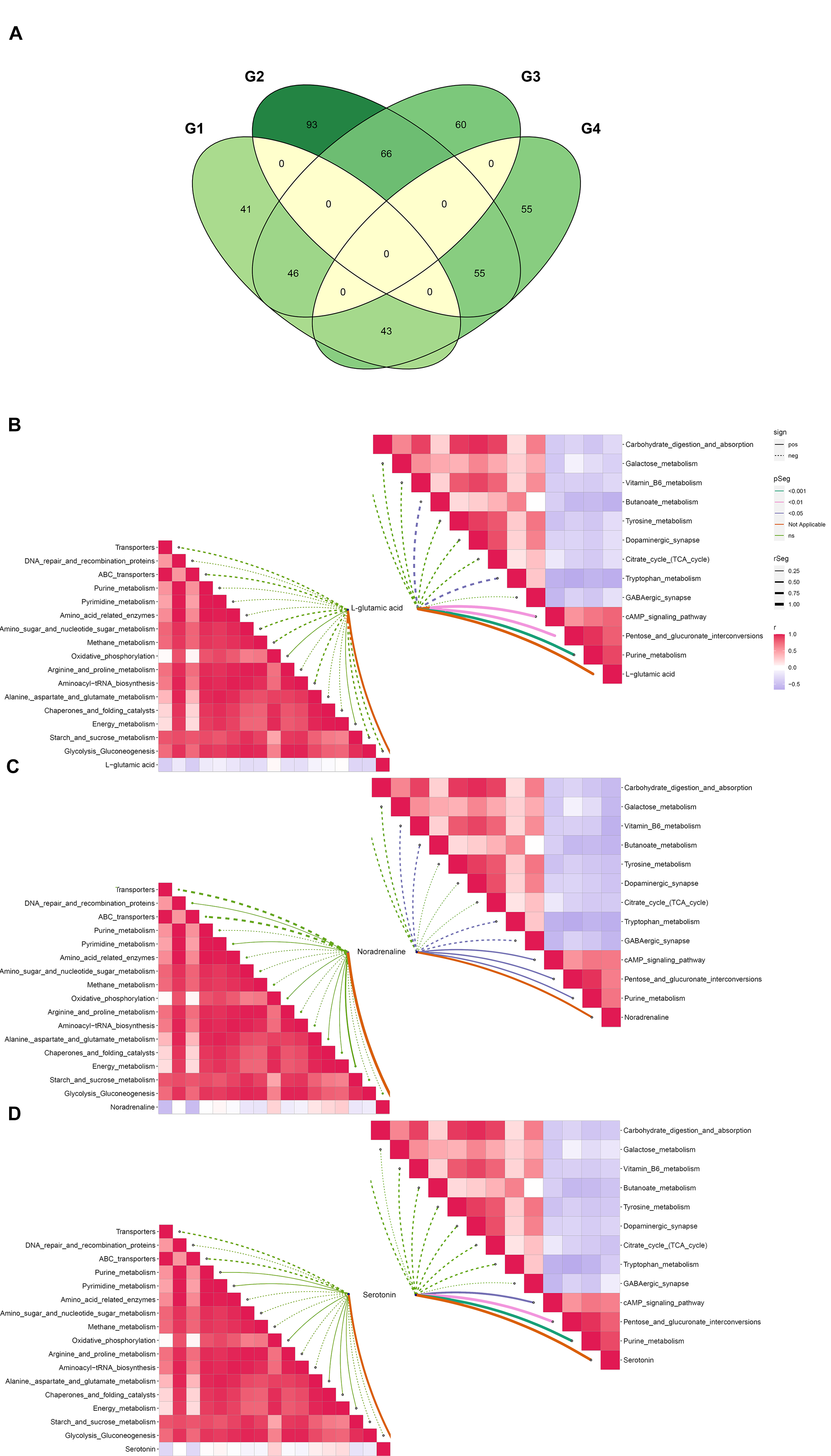

Supplement: Supplementary Figure 3 — Fecal microbiota transplantation (FMT) did not change the morphometry and function of the cardiac tissue. (A,B) Cardiac size and cardiac fibrosis. (C) Ejection fraction (EF). (D) Left ventricular posterior wall dimensions at end-diastole (LVPWd). (E) Left ventricular internal diameter diastole (LVIDd). (F) Serum brain natriuretic peptide (BNP) levels. (G) Relative heart weight. (H) BW. n = 6. [file Image_3.TIF]

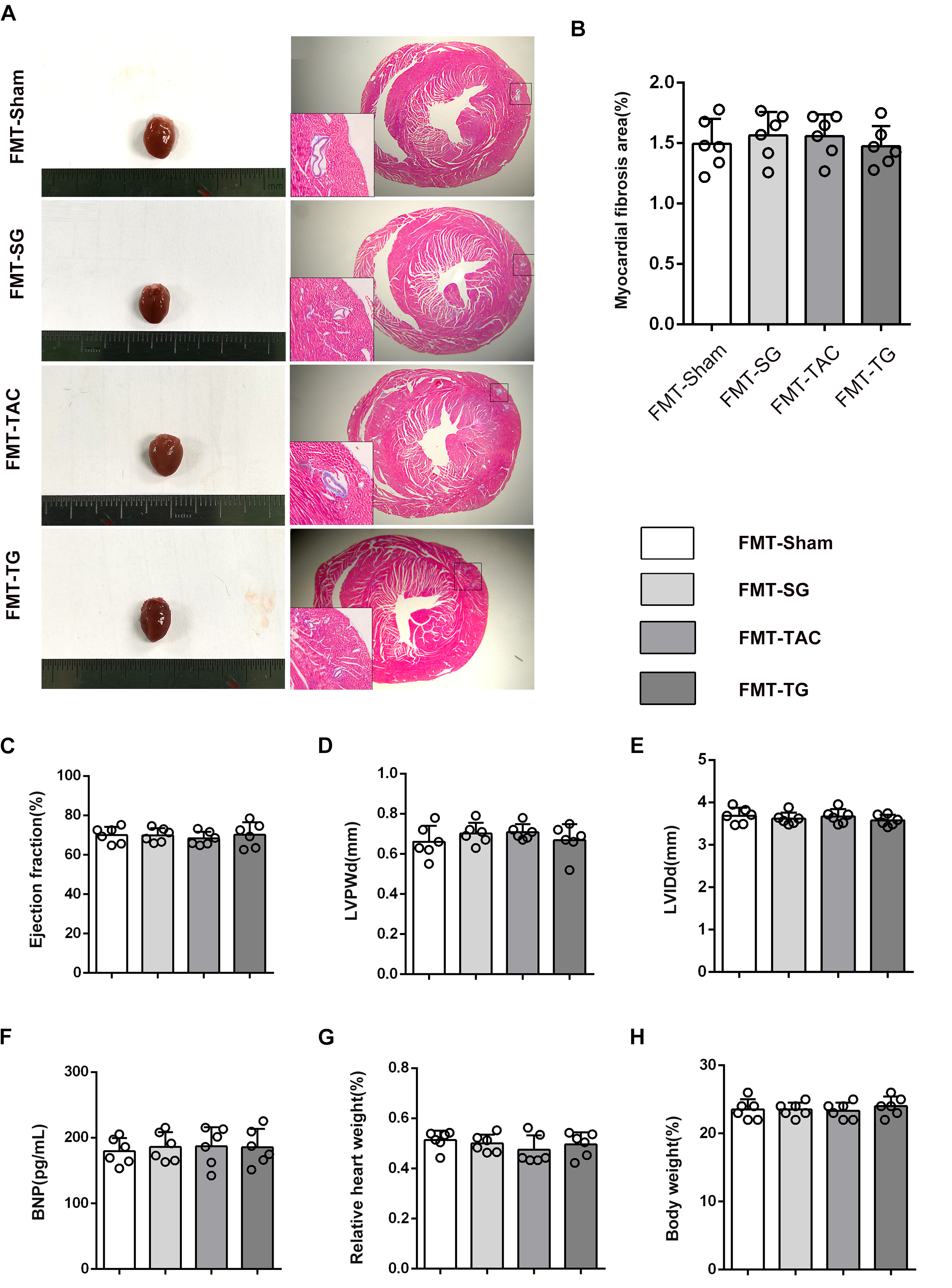

Supplement: Supplementary Figure 4 — The predicted functions of the shared upregulated metabolism in fecal and serum samples. (A). Shared upregulated metabolism in fecal and serum samples. (B) L-Glutamic acid enrichment pathway. (C) Noradrenaline. (D) Serotonin. Left, fecal; right, serum. [file Image_4.TIF]
